# Retrospective characterization of seizure semiology and treatment using continuous video‐EEG monitoring in neonatal encephalopathy in Uganda

**DOI:** 10.1002/epd2.20299

**Published:** 2024-11-18

**Authors:** J. Proietti, C. Nanyunja, S. R. Mathieson, E. Duckworth, S. Sadoo, I. Mambule, A. Nakimuli, C. J. Tann, G. B. Boylan

**Affiliations:** ^1^ INFANT Research Centre University College Cork Cork Ireland; ^2^ Department of Engineering for Innovation Medicine University of Verona Italy; ^3^ Department of Maternal and Child Health AOUI Verona (full member of the European Reference Network EpiCARE) Italy; ^4^ MRC/UVRI and LSHTM Uganda Research Unit Entebbe Uganda; ^5^ London School of Hygiene and Tropical Medicine London UK; ^6^ University College London Hospitals NHS Trust London UK; ^7^ Kawempe National Referral Hospital Kampala Uganda; ^8^ Makerere University Kampala Uganda

**Keywords:** neonatal encephalopathy, neonatal seizures, seizure semiology, Uganda

## Abstract

**Objective:**

Neonatal encephalopathy (NE) is a leading cause of childhood death and disability, particularly in sub‐Saharan Africa. Detection of NE‐related seizures is challenging. We explored NE seizure semiology and management in Uganda.

**Methods:**

Video‐EEG was recorded (days 1–5), seizure semiology reviewed according to ILAE classification and administration of antiseizure medication (ASM) evaluated. Clinicians treated seizures based on the clinical presentation alone.

**Results:**

Among 50 participants, 52% (26) had EEG‐confirmed seizures; 70% (18) combined electroclinical/electrographic; 4% (1) exclusively electroclinical; 22% (6) electrographic. Of those with electroclinical seizures (19), 42% displayed >1 semiology. Distribution of seizure semiology was; clonic 34% (11); autonomic 24% (8, of which 6 had prolonged ictal apnea); automatisms 18% (6); behavioral arrest 12% (4); and sequential 12% (4). ASM was administered to 64% (32/50). Of those with EEG‐confirmed seizures, only 62% (16/26) received ASM. In the non‐seizure group, 38% (9/24) received ASM during monitoring. ASM was administered 42 times, of which 45% (19) were considered appropriate.

**Significance:**

In this Ugandan NE population, incidence of seizures was high and clinical manifestations frequent. Clonic, autonomic and automatisms were most common. Clinical management was challenging, with both under and overtreatment evident. Respiratory impairment due to autonomic seizures frequently went unrecognized and is a prominent concern, particularly in settings without neonatal intensive care.

**Key points**

- Intrapartum‐related neonatal encephalopathy is a leading cause of early childhood morbidity and mortality, with the highest burden in sub‐Saharan Africa.
- Understanding seizure semiology of NE‐related seizures is important to inform understanding of clinical presentation and prompt seizure management.
- In this Ugandan neonatal encephalopathy cohort, of those with EEG confirmed seizures, most presented with clinical manifestations: clonic, autonomic and automatisms were the most common seizure types observed.
- Clinical diagnosis of seizures proved difficult with both under and over treatment evident.
- Respiratory impairment due to autonomic seizures frequently went unrecognized as seizure activity and emerged as a prominent concern in this setting with limited access to neonatal intensive care.

## 1 INTRODUCTION

Intrapartum‐related neonatal encephalopathy (NE) affects over a million term newborns each year globally and is a substantial contributor to under 5 mortality and long‐term morbidity.^1^, ^2^ The majority of infants affected by NE are born in low‐ and middle‐ income countries (LMICs) where neonatal intensive care and neuroprotective interventions such as therapeutic hypothermia are not available.^3^, ^4^ Seizures represent an important risk factor for adverse outcome after NE,^5^ however, prompt detection and treatment of NE‐related seizures can be challenging.

Continuous video‐electroencephalographic (cEEG) monitoring remains the gold standard investigation for the early assessment of brain injury severity and detection of seizures. However, the need for specialized equipment and related technical challenges mean that it remains largely unavailable in LMIC settings.^6^ As a result, acute provoked seizures occurring secondary to brain injury are at risk of being undetected or misdiagnosed, and hence not adequately treated, as shown in high‐income countries (HICs).^7^ In the absence of cEEG, identification of seizures relies solely on clinical diagnosis.^8^, ^9^, ^10^ For this reason, there is an urgent clinical need to understand how seizures manifest among neonates with NE in LMIC settings. To date, most studies focusing on the semiology of neonatal seizures in NE have been conducted in HICs, including the recently updated seizure classification for neonates.^11^, ^12^

Between 2019 and 2022, a facility‐based NE cohort was established at Kawempe National Referral Hospital in Kampala, Uganda to explore the etiology, clinical course and early childhood outcomes after NE in a LMIC sub‐Saharan African setting. The “Baby BRAiN” study aims to enhance understanding of NE and inform future research on neuroprotective strategies in settings where therapeutic hypothermia is not routinely practiced.^13^ As part of the study, recruited neonates received cEEG to examine electrographic brain activity and seizure burden, providing the opportunity to examine seizure semiology and management among neonates with NE in the Ugandan setting. The primary aim of this sub‐study was to explore the electroclinical semiology of neonatal seizures among NE infants receiving continuous multichannel video‐EEG monitoring in Uganda, as per the new ILAE classification of neonatal seizures. In addition, we aimed to understand the relationship between seizures that were clinically suspected and treated at the cotside and retrospective analysis of simultaneous video‐EEG annotated seizures.

## 2 METHODS

### 2.1 Study setting and participants

Kawempe National Referral Hospital is located in Kampala, Uganda's capital city. It receives high‐risk referrals from across the city, delivering around 25 000 women each year with around 300–350 neonatal admissions for moderate‐severe encephalopathy each year [unpublished data]. Routine care during the sub‐study period included simple continuous positive airway pressure ventilation, intravenous fluids including glucose, antibiotics and first line anti‐seizure medication (ASM). Therapeutic hypothermia is not available, and care for NE infants is largely supportive. Facilities for continuous intrapartum fetal monitoring or cord/neonatal blood gas measurement are not routinely available. Between October 2019 and October 2020, infants with NE admitted to the neonatal unit were recruited to the “Baby BRAiN” study. Recruitment was limited by the suspension of all research in Uganda between March and July 2020, due to SARS‐CoV‐2 outbreak and related lockdown, and by availability of cEEG equipment. Inclusion criteria for this observational cohort study were: ≥36 weeks' gestation on Ballard assessment; age <48 h at time of recruitment; birth weight ≥1.8 kg; need for continued resuscitation at birth and/or Apgar score ≤5 at 5 min; clinical evidence of NE (defined as Thompson score ≥5)^14^; and parental informed written consent. The Thompson score is a numeric scoring system to assess the severity of perinatal asphyxia, derived from nine aspects of the neurological examination (tone, level of consciousness, seizures, posture, Moro reflex, grasp reflex, suck reflex, fontanelle findings and respiration); the total score ranges from 0 (normal) to 22. Exclusion criteria included; neonates with major congenital malformations, absent heart rate at 10 min and those where death was felt to be imminent, and mother living permanently >20km away from the hospital. Relevant clinical and survival outcome data were collected locally by trained research personnel and included the presence of presumed clinical seizures based on abnormal movements noted by bedside staff. Timing and type of ASM administered were also collected.

### 2.2 EEG recording and seizure analysis

cEEG was applied and maintained by clinical research staff supervised by the research coordinator (CN). Equipment and training regarding EEG application and EEG system function was provided by ED and CT supported by neurophysiology experts from the INFANT research center, University College Cork, Ireland. Multichannel video‐EEG was commenced as soon as possible after birth once consent was given, and was continuously recorded up to the fifth day of life when possible, using the portable Lifelines EEG systems (Lifelines iEEG, UK). Electrodes were positioned at F3, F4, C3, C4, Cz, T3, T4, O1 and O2 according to 10–20 EEG electrode placement system adapted for the neonates. Recording and review settings included a bipolar montage (F4‐C4, C4‐O2, F3‐C3, C3‐O1, T4‐C4, C4‐CZ, CZ‐C3, C3‐T3), high pass filter .5 Hz, low pass filter 70 Hz, notch filter, sensitivity 7‐10 μV/mm, timebase 15–20 mm/s. Single channel electrocardiography and respiration monitoring synchronized with the EEG trace were also recorded. The video‐EEG was uploaded, subject to network availability, to a cloud‐based server with INFANT center personnel able to provide signal quality assessment and technical feedback to the local team. EEG monitoring is not routinely available outside tertiary intensive care settings in high‐income country settings and is not currently available in any government facility in Uganda. Clinically recognized seizures were treated based on clinical diagnosis, as per local routine practice and according to local protocols for anti‐seizure medication administration. As EEG interpretation is recognized to be a highly specialized skill with risk of harm if interpreted incorrectly, the video‐EEG screen was covered. Due to the challenges with timely uploading of raw EEG data, it was not possible to provide continuous 24‐h feedback to the clinical team, however in the event that the cEEG feed was able to be viewed live, any concerns were communicated to the principal investigator.

The type, dose and timing of every dose of ASM administered to each neonate were recorded.

The analysis of each recording was performed retrospectively by one member of a group of 3 experienced neonatal neurophysiologists (JP, SRM, GBB), blind to clinical information. All seizures were annotated according to a standard protocol: a seizure was defined as a sudden repetitive, stereotyped discharge of minimum 10 s' duration on one or more EEG channel with evolving frequency, amplitude and morphology.^15^ Status epilepticus was defined as the presence of more than 30 min of seizures in a 1‐h epoch.^16^

For each neonate, all video‐EEG segments annotated as seizures were selected and further reviewed for seizure semiology analysis. Seizure types were assigned according to the recently updated ILAE classification of neonatal seizures, based on electroclinical phenotype.^17^ Any electrographic seizure seen on EEG that was not associated with evident clinical signs was defined as electrographic‐only. An electro‐clinical seizure was defined as definite clinical signs simultaneously coupled with an electrographic seizure. Seizure types were described by their predominant clinical features. Automatisms were characterized by non‐purposeful, stereotyped, and repetitive motor activity such as orolingual movements, or movements of the limbs like cycling. Clonic seizures were characterized by regularly repetitive jerking, either symmetric or asymmetric, involving the limbs, head or trunk. Tonic seizures showed sustained increase in muscle contraction, focal, unilateral, or bilateral. Autonomic seizures were defined as distinct alteration of autonomic nervous system function (i.e. color, breathing pattern, heart rate). Behavioral arrest seizures were defined as pause of activities, freezing, immobilization. Sequential seizures, newly added to ILAE classification of neonatal seizure types, described seizure events with a sequence of signs, symptoms, and EEG changes at different times, in which no predominant feature could be determined. In some cases, the video could not be used due to poor lighting in the neonatal unit and/or poor‐quality images, and, in this event, the seizure was reported as unclassified.

Although standardized guidance on how soon seizures should be treated is currently lacking, for the purpose of this study, we anticipated that a seizure episode should be treated within 1 h of onset if recognized.^18^ The time of administration of ASM loading dose was compared with that of seizures retrospectively annotated on the video‐EEG recordings. The administration of either phenobarbital or phenytoin was assessed. Maintenance doses, administered in some cases, were not included in our analysis. Appropriately managed seizures were defined as EEG confirmed seizures treated within 60 min of onset. Treatment failure was defined as EEG confirmed seizure not followed by treatment administration. Paroxysmal non‐epileptic events occurring in the 60 min preceding an ASM load, or concomitant to electrocardiography and/or respiration monitoring alterations, were also examined.

### 2.3 Statistical analysis

Baseline characteristics of the cohort were summarized with counts (percentages) for categorical variables, mean (standard deviation [SD]) for normally distributed continuous variables or median (IQR) for other continuous variables. Seizures reported and treated by bedside staff and retrospectively video‐EEG recognized seizures were reported as counts and percentages. The burden of each semiology seizure type was reported according to the number and proportion of seizures for each participant.

## 3 RESULTS

### 3.1 Baseline characteristics

A total of 51 neonates with NE were recruited, and 50 had video‐EEG data of diagnostic quality. The demographics and baseline EEG characteristics of the 50 patients included in the analysis are described in Table 1.

**TABLE 1:** Clinical and EEG characteristics of the 50 patients included in the analysis.

Characteristics	*N* = 50
Clinical characteristics	
Sex, male	66% (33)
Gestational age, weeks	38 (38‐40) [36–42]
Birthweight, grams	3117 (451) [2300–4150]
5‐min Apgar score*	6 (4.5‐7) [0–10]
Age of recruitment, hours	19 (11‐24.3) [3.1–46.1]
NE severity (Sarnat grade)	
Mild	24% (12/50)
Moderate	30% (15/50)
Severe	42% (21/50)
EEG characteristics	
Age at start of monitoring, hours	21.1 (14.2‐29.8) [5.4–48.8]
Duration of continuous video‐EEG, hours	71.4 (52.4‐72.2) [10.3–75.3]
Confirmed electrographic seizures, % (*n*)	52% (26/50)
Episodes of status epilepticus, % (*n*)	50% (13/26)
Neonatal death, % (*n*)	26.7% (12/46)

*Note*: Birthweight is reported as mean (SD) [range], all other metrics are median (IQR) [range] or % (*n*).

* Missing data for 2 participants.

Continuous video‐EEG (cEEG) was recorded for a median (IQR) duration of 71.4 (52.4‐72.2) h. Monitoring commenced within 48 h of age in all neonates, within 24 h in 76% (38/50), and continued for at least 24 h in 92% (46/50). Of the 50 neonates, 26 (52%) had video‐EEG confirmed seizures identified by the expert group. Of the 26 neonates, 13 (50%) had periods of status epilepticus.

### 3.2 Seizure semiology

**FIGURE 1:**
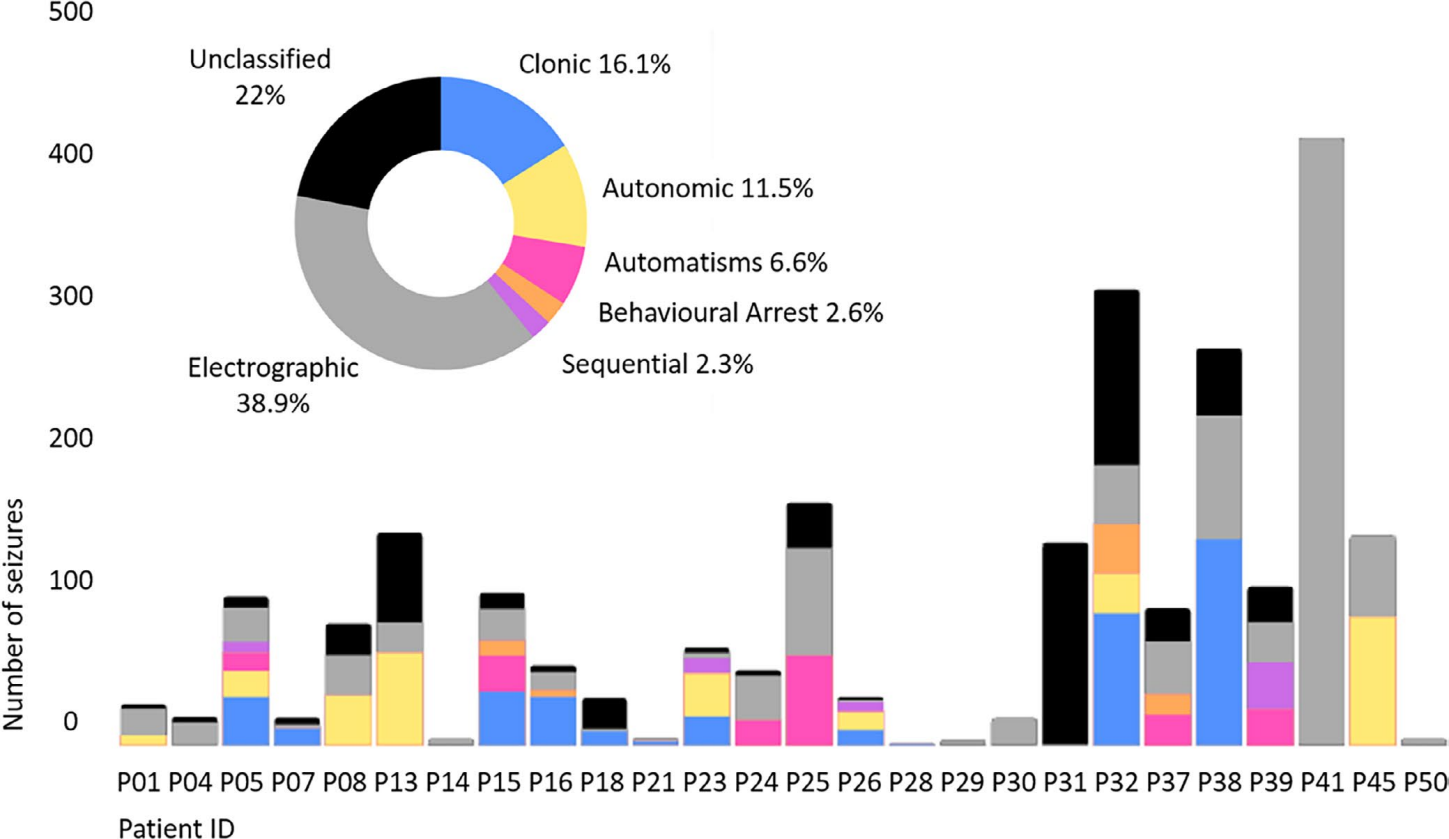
Distribution of seizure types in the cohort (circle, percentage of the total number of seizures) and in single patients (columns, number of seizures).

On video‐EEG analysis, 70% (18/26) of neonates with seizures had a combination of electroclinical and electrographic seizures, 4% (1) had exclusively electroclinical seizures and 22% (6) had only electrographic seizures. 4% (one patient) had exclusively seizures classified as unknown due to a malfunction in video recording. Among patients in which a video‐polygraphy clinical correlate was synchronous with the EEG discharge in at least one part of the seizure, 58% (11/19) displayed one seizure type and 42% (8/19) more than one seizure type (2 in three, 3 in four, 4 in one). The distribution of all seizure semiology types was as follows: clonic 34% (11), autonomic 24% (8), automatisms 18% (6), behavioral arrest 12% (4), and in 12% of cases (4) electroclinical manifestations consistent with the definition of sequential seizures. The latter were prolonged in duration and characterized by the succession of distinct ictal patterns on the EEG associated with different clinical manifestations, without a predominance of one or the other being recognizable; clonic, automatisms and autonomic manifestations were more frequently part of sequential seizures. Six of the 8 neonates with autonomic seizures had prolonged ictal apnea. Among the 18 neonates displaying a combination of electroclinical and electrographic seizures, the mean (SD) percentage of electroclinical seizures was 52% (30) and that of electrographic seizures was 24% (25). A number of unclassified seizures were also present in 16 out of 18 neonates in this group (mean 18%, SD 17). The distribution of seizure types in the cohort and in single neonates is summarized in Figure 1.

Of 13 neonates with status epilepticus, 84% (11) had a combination of electroclinical and electrographic seizures, 8% (1) had unknown seizures, and 8% (1) had only electrographic seizures (brief repetitive focal discharges never exceeding a low‐medium voltage). In the remaining 13 neonates (without status epilepticus), 54% (7) had a combination of electroclinical and electrographic seizures, 8% (1) had only electroclinical seizures and 38% (5) had only electrographic seizures.

### 3.3 Management with anti‐seizure medications

**FIGURE 2:**
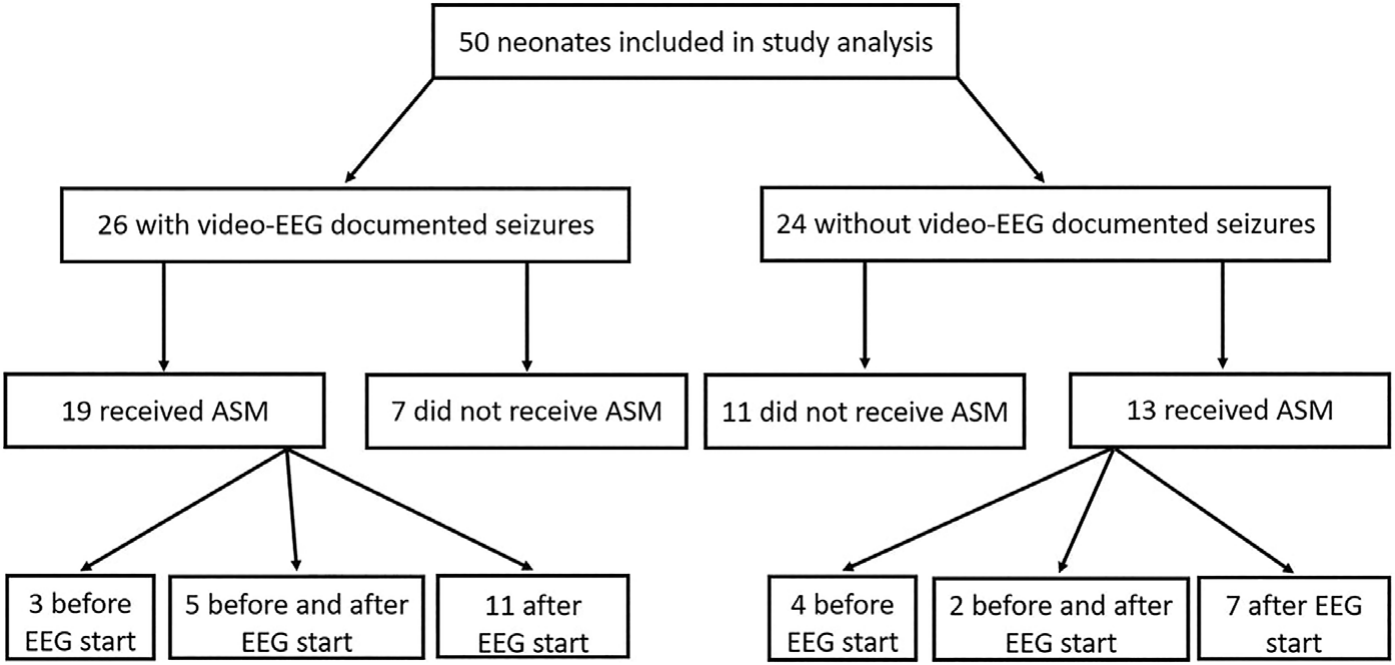
Flow diagram seizures and antiseizure medications (ASM) use in the cohort.

Information about seizures and ASM used in the cohort are summarized in Figure 2. Overall, 64% (32/50) of neonates received ASMs either during or prior to EEG starting. All received phenobarbitone as their first‐line treatment (dosage between 10 and 20 mg/kg). Of those receiving ASM, 25 (78%) received at least one additional ASM bolus or hemi bolus of either phenobarbital or phenytoin, and 14 (44%) received their first ASM before the video‐EEG commenced. Seven neonates received treatment exclusively before the monitoring started: in 4, subsequent EEG recording did not document seizures, while the remaining 3 displayed later seizures.

**FIGURE 3:**
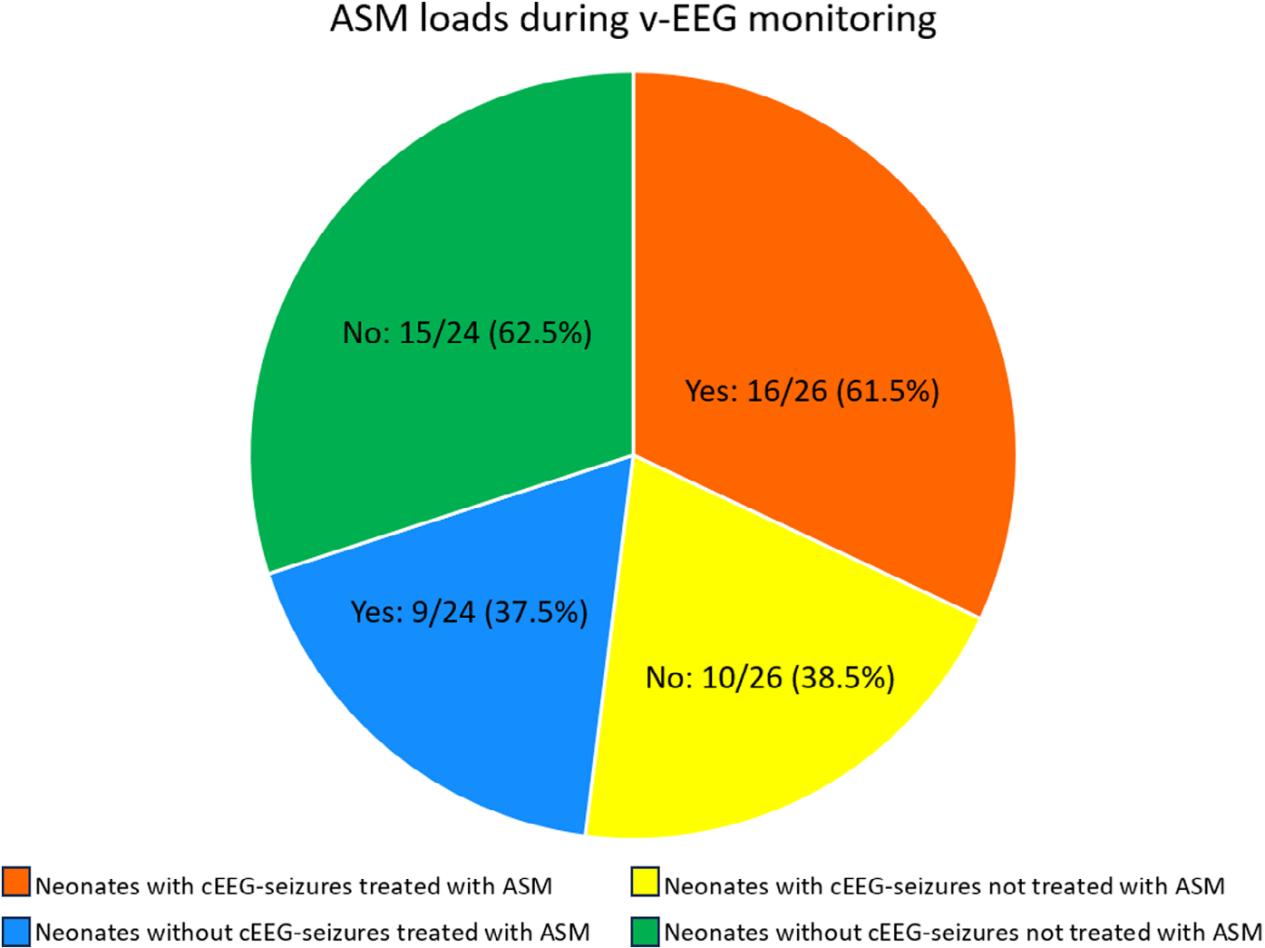
ASM loads administered during the monitoring period in patients with and without video‐EEG recorded seizures.

ASM loading doses were received by 50% (25/50) of neonates during continuous video‐EEG recording. Of those with EEG‐confirmed seizures, only 16/26 (62%) received treatment during EEG monitoring (including 5 neonates who also received ASM before the start of EEG monitoring), and 7 out of 26 (27%) neonates with EEG documented seizures never received an ASM at any time. Nine out of 24 (38%) in the non‐seizure group were also treated. The administration of ASM loads during the monitoring period in neonates with and without video‐EEG recorded seizures is summarized in Figure 3. In total, ASM loading doses were administered 42 times after recording commencement: 15/42 (36%) in the group without video‐EEG‐documented seizures (thus by definition inappropriate) and 27/42 (64%) in the group with video‐EEG documented seizures. Among the latter, in relation to the presence or absence of seizures in the 60 min prior to the ASM administration, 19 (45%) were appropriate and 8 (19%) non appropriate. Twelve neonates had at least one episode treated appropriately. The number of ASM loads received after EEG commencement ranged from 1 to 4 (median 2, IQR 1–2). In 7 patients, the first ASM load was appropriately administered during monitoring, after a median (IQR) time of 8.0 (3.8–8.9) h from the first video‐EEG documented seizure.

In the subgroup of patients with video‐EEG documented status epilepticus, 1/13 (8%) received treatment exclusively before the beginning of the recording, 2/13 (15%) were never treated, and 10/13 (77%) patients received ASM while the monitoring was ongoing (including 3 who also received ASM before the start of EEG monitoring). Of the 15 ASM loads administered during EEG monitoring in this subgroup, 13 were appropriate and 2 inappropriate.

ASM administration, seizure semiology and seizure types for each patient are summarized in Table 2. Video‐EEGs of seizures recorded before the administration of any ASM was available in 11 patients, and 8 had a clinical component associated with their seizures prior to treatment. Of the 19 adequately treated seizures, 8 were clonic, 5 autonomic, 1 automatisms, 2 sequential and 3 had unknown semiology.

**TABLE 2:** ASM administration, seizure semiology and burden of individual seizure type expressed in numbers and percentage in neonates with video‐EEG‐documented seizures (*n* = 26).

	Status epilepticus (*n* = 13)	Never received ASM (*n* = 7)	Received ASM before start of cEEG (*n* = 9)	Received ASM after start of cEEG (*n* = 16)	Electroclinical seizures (*n* = 19)						Electrographic (*n* = 25)	Unclassified* (*n* = 18)
					Clonic (*n* = 11)	Autonomic (*n* = 8)	Automatisms (*n* = 6)	Behavioral arrest (*n* = 4)	Sequential (*n* = 4)	Total		
P01	No	–	X	X	–	7 (24%)	–	–	–	7 (24%)	19 (66%)	3 (10%)
P04	No	–	–	X	–	–	–	–	–	–	16 (80%)	4 (20%)
P05	Yes	–	X	–	35 (33%)	19 (18%)	13 (12%)	–	8 (7%)	75 (70%)	25 (23%)	8 (7%)
P07	No	X	–	–	12 (60%)	–	–	–	–	12 (60%)	3 (15%)	5 (25%)
P08	Yes	–	–	X	–	36 (41%)	–	–	–	36 (41%)	29 (33%)	23 (26%)
P13	Yes	–	–	X	–	67 (44%)	–	–	–	67 (44%)	22 (14%)	65 (42%)
P14	No	X	–	–	–	–	–	–	–	–	4 (100%)	–
P15	Yes	–	X	X	39 (35%)	–	26 (23%)	11 (10%)	–	76 (68%)	23 (21%)	12 (11%)
P16	Yes	–	–	X	35 (60%)	–	–	5 (9%)	–	40 (69%)	13 (22%)	5 (9%)
P18	No	–	X	–	10 (29%)	–	–	–	–	10 (29%)	2 (6%)	22 (65%)
P21	No	–	X	X	3 (60%)	–	–	–	–	3 (60%)	2 (40%)	‐
P23	Yes	–	X	X	21 (29%)	31 (44%)	–	–	11 (15%)	63 (88%)	4 (6%)	4 (6%)
P24	No	–	X	–	–	–	18 (33%)	–	–	18 (33%)	32 (59%)	4 (8%)
P25	Yes	X	–	–	–	–	65 (37%)	–	–	65 (37%)	78 (44%)	33 (19%)
P26	Yes	–	–	X	11 (31%)	13 (37%)	–	–	7 (20%)	31 (88%)	2 (6%)	2 (6%)
P28	No	X	–	–	1 (100%)	–	–	–	–	1 (100%)	–	–
P29	No	X	–	–	–	–	–	–	–	–	3 (100%)	–
P30	No	–	–	X	–	–	–	–	–	–	19 (100%)	–
P31	Yes	X	–	–	–	–	–	–	–	–	–	147 (100%)
P32	Yes	–	X	X	96 (29%)	29 (9%)	–	–	–	–	43 (13%)	128 (38%)
P37	Yes	–	X	–	–	–	22 (22%)	15 (16%)	–	37 (38%)	38 (38%)	24 (24%)
P38	No	–	–	X	150 (52%)	–	–	–	–	150 (52%)	90 (31%)	49 (17%)
P39	Yes	–	–	X	–	–	26 (23%)	–	–	–	–	26 (23%)
P41	Yes	–	–	X	–	–	–	–	–	–	–	–
P45	No	–	–	X	–	93 (61%)	–	–	–	93 (61%)	59 (39%)	–
P50	No	X	–	–	–	–	–	–	–	–	4 (100%)	–

* Seizures associated with poor quality video.

**FIGURE 4:**
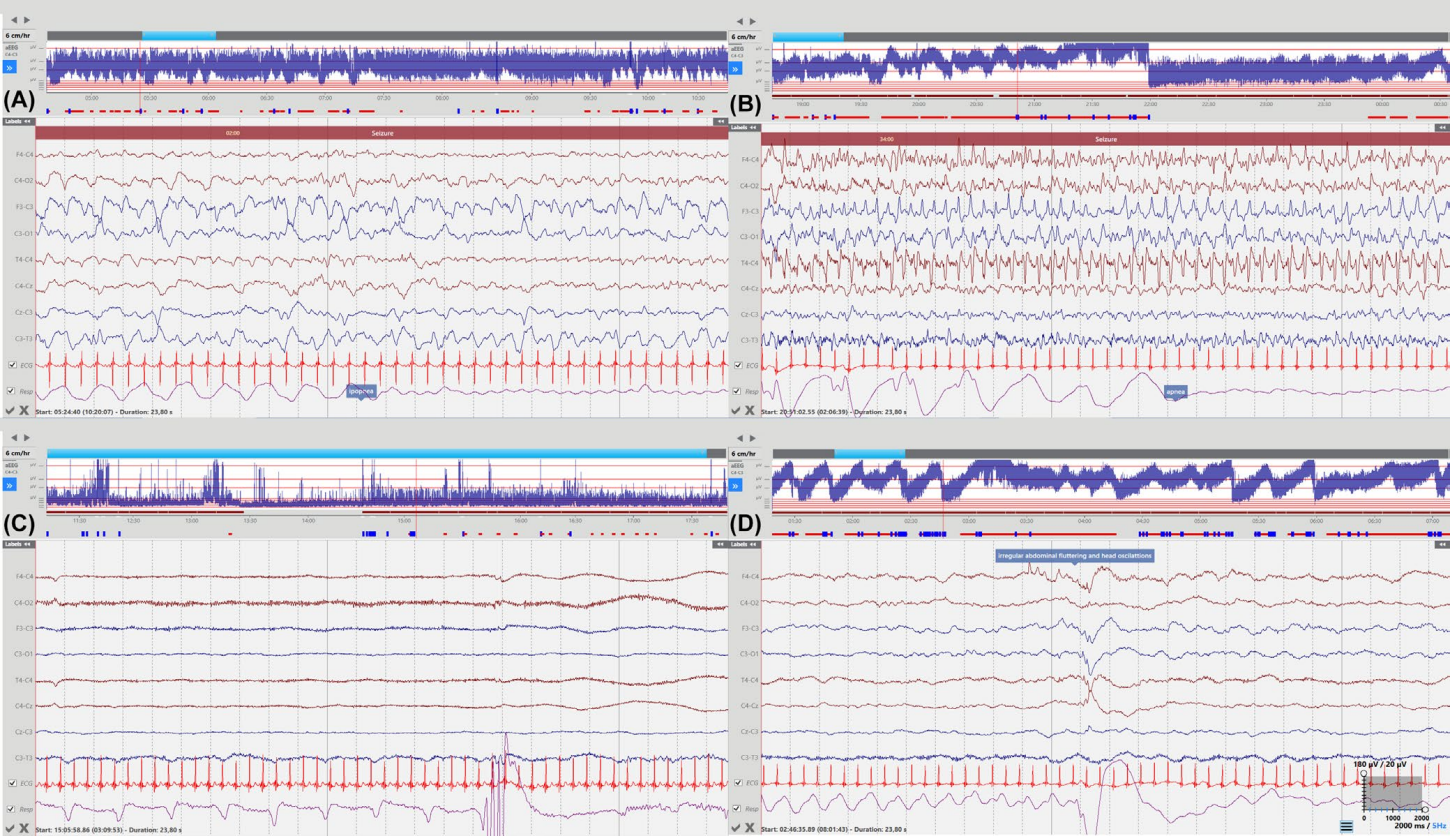
Apnea occurring during autonomic seizures in patients 13 (A) and 26 (B) and non‐epileptic abdominal flutter in patients 1 (C) and 26 (D) evident on the polygraphy channel exploring thoracoabdominal shifts. Filters settings are shown in (D).

The analysis of the video‐polygraphy recording in the 60 min preceding inappropriate administration of ASM loading doses (23 in total) revealed the presence of generalized tonic extensor posturing events without clear asymmetry in 6 cases, jerky non‐rhythmic movements during crying in 4 cases, nonepileptic oral automatisms in 3 cases, paroxysmal nonepileptic tremor in 1 case; these conditions were presumably interpreted as ictal by the local clinical team. Nonepileptic oral automatisms were sucking movements in all cases, not clearly distinguishable clinically from those observed in the context of electroclinical events classified as automatisms seizures (a minority of the latter, namely in two patients, were associated however with repetitive cycling movements which were not observed in events without EEG ictal correlate). In addition, 12 neonates (8 in the group with video‐EEG documented seizures and 4 in the non‐seizure group) also presented with abnormal repetitive abdominal movements or gasping, sometimes showing pseudo periodic evolution, unrelated to ASM administration; this pattern was never associated with concomitant modifications of the EEG tracings. Illustration of apnea occurring during autonomic seizures and of the non‐epileptic abdominal flutter or gasping is provided in Figure 4.

## 4 DISCUSSION

This study is the first, to our knowledge, to describe seizure semiology on continuous video‐EEG monitoring from a LMIC setting in sub‐Saharan Africa and provides evidence of feasibility in this Ugandan research setting. The findings reveal the high incidence of seizures with video‐EEG documented seizures present in over half of neonates monitored, and over a quarter of all neonates, meeting the criteria for status epilepticus. Clonic, autonomic and automatisms were the most frequently encountered seizure types seen. Clinical diagnosis of seizures proved difficult, with both under and overtreatment evident. Clonic seizures were the most reliably diagnosed on clinical evaluation alone. Of particular concern was autonomic seizures manifesting as ictal apnea which occurred in close to a quarter of participants with seizures and were frequently unrecognized as seizures by clinical staff.

There is an urgent need to further our understanding of neonatal seizures among infants with NE in LMICs, to optimize management and outcomes. Very few centers in Africa (outside South Africa) currently offer therapeutic hypothermia and evidence to date supporting its safety or efficacy in LMICs is lacking. Recently, the HELIX trial in India raised substantial safety concerns with increased mortality among cooled neonates.^4^ A meta‐analysis on therapeutic hypothermia for NE in LMICs did not demonstrate a significant difference in the risk of death (although highlighting higher rates of thrombocytopenia and hemorrhage), but also showed little to no difference in clinical outcomes between treated and untreated neonates.^19^ Given increasing awareness in HIC settings that high seizure burden contributes to neurodevelopmental impairment,^5^ prompt detection and adequate treatment of seizures in NE may contribute to neuroprotective strategies to improved outcome in LMICs. In our Ugandan cohort, the prevalence of status epilepticus was common, occurring in around half of all neonates experiencing seizures. This is similar to the prevalence reported in NE cohorts without therapeutic hypothermia from HICs and is substantially higher than the rate of status epilepticus reported in cooled NE cohorts in HICs.^20^, ^21^ However, published studies aimed at improving diagnosis and management of neonatal seizures almost exclusively originate from HICs, with, most studies from LMICs relying on clinical diagnosis alone for seizure identification.^22^

The video‐EEG analysis of seizure semiology performed according to the updated ILAE classification of seizures in the neonate has provided novel insights regarding neonatal seizures in a LMIC. Indeed, the aim of the ILAE taskforce who developed this classification was to ensure that this scheme was applicable to all healthcare settings. Electroclinical seizures were more common in this Ugandan cohort compared to other similar cohorts in HICs after the advent of therapeutic hypothermia. The ILAE task force, who proposed the new classification, described 19 electrographic, 5 clonic, 3 sequential and 2 tonic seizures in a sub‐cohort of 29 neonates with hypoxic‐ischemic encephalopathy (HIE) and seizures; similarly in a double‐cohort study across USA and Belgium, 15 of 26 HIE neonates never had a clinical correlate to their seizures.^18^, ^23^, ^24^

Clonic, autonomic and automatisms were the more frequently encountered seizure types in this Ugandan cohort, with clonic seizures the more reliably diagnosed on the basis of clinical evaluation only.^25^ Seizures manifesting as respiratory impairment only were common (75% of neonates with autonomic seizures and 23% of the total number of patients with seizures had prolonged ictal apnea). Rhythmic high amplitude spike‐and‐waves, diffuse or predominant over the central derivations, were commonly observed in association to clonic seizures. No reproducible EEG pattern was observed in other seizure types, and particularly no specific EEG discharge morphology or localization was identified related to ictal apneas. Some neonates showed non‐ictal repetitive abdominal movements or gasping, sometimes showing pseudo periodic evolution and resembling a diaphragmatic flutter.^26^ Neither epileptic spasms nor myoclonic seizures were seen, as expected in neonates with NE.^18^ Tonic seizures were not encountered; a focal increase in muscle contraction was only observed in the context of clonic manifestations. Electrographic only seizures were present in almost every neonate with seizures but represented the only seizure type in six patients.

Clinical diagnosis of seizures in this study proved difficult, with both under and overtreatment evident. This is in line with data on staff identification of seizures in HICs, where both underdiagnosis due to missing discrete seizure manifestations and electrographic only seizures, and overdiagnosis due to misdiagnosing abnormal nonepileptic movements as seizures, have been highlighted.^7^, ^27^ It is now well established that EEG is essential for seizure detection, and clinical diagnosis alone is not reliable.^17^ During periods of EEG monitoring, 38% of neonates without any cEEG evidence of seizures were given ASM, and 38% with cEEG evidence of seizures were not treated. While on the one hand, untreated seizures may add to any pre‐existing brain injury and alter seizure thresholds in the brain; on the other hand, inappropriate ASM administration exposes the newborn to potential ASM‐related drug toxicity and complications such as sedation and respiratory depression, which is particularly relevant in low‐resource settings where mechanical ventilation is unavailable. A few neonates may have responded to the ASM administered before monitoring started, given that 4 were given an ASM before the commencement of cEEG monitoring and then never went on to have EEG‐documented seizures. Respiratory impairment emerged as a prominent concern, both during seizures and at other times. Seizures manifesting as respiratory impairment only were common and frequently unrecognized as seizures by clinical staff. No specific EEG discharge morphology or localization was identified related to ictal apnea. Some neonates showed non‐ictal repetitive abdominal movements or gasping, sometimes showing pseudo periodic evolution and resembling a diaphragmatic flutter.^26^ An increased awareness of the different manifestations of seizures in this setting might help local clinicians recognize seizures. However, the presented semiology analysis was based on video‐EEG recordings and conducted by neurophysiologists with specific expertise in neonatal EEG and seizure semiology interpretation. Epileptic and nonepileptic clinical manifestations in critically ill patients will probably remain difficult to identify for healthcare workers without specialized training and without the support of EEG.

The findings of this pilot observational study clearly highlight the potential benefit of introducing context specific and targeted EEG monitoring in this setting to effectively manage seizures, with either training in interpretation for local clinical staff or potentially remote expert surveillance. A system of neonatal neurocritical care delivered at remote limited resource hospitals through telehealth was recently developed in Brazil, where newborns with HIE receiving TH from 32 Hospitals were remotely monitored with amplitude‐integrated electroencephalography and video imaging, and bedside clinicians received real‐time assistance from trained neonatologists and neurologists.^28^ Following in the footsteps of Variane and collaborators, the development and dissemination of simple and innovative models for neurophysiology monitoring in neonates in low‐resource settings should be prioritized.

The presence of seizures in four patients that were consistent with the definition of sequential seizures (seizure presenting with a variety of clinical signs occurring in a sequence without a predominant feature determined) is interesting. According to the existing definition, sequential seizure may be present in some infants with HIE although they are more commonly seen in other etiologies such as self‐limited neonatal epilepsy or neonatal onset epileptic encephalopathy.^18^ Seizures in this Ugandan cohort were prolonged and characterized by a sequence of clinical features coinciding with the slow evolution in amplitude and morphology of the discharge, appear to be clearly different compared to the briefer and stereotyped trains of electroclinical manifestations typically seen in genetic etiologies. Different interpretations are also evident in recently published studies which have applied the new classification to neonatal populations: de Correa et al. reported sequential seizures in all etiology groups in their cohort and mainly in HIE, while Cornet et al. identified sequential seizures specifically in 11 of the 18 patients with confirmed channelopathies within a cohort also including a larger number of neonates with seizures due to stroke or HIE.^24^, ^29^ The latter, additionally, recognized tonic features as the most specific for the genetic etiology, whether representing the initial phase of sequential seizures or constituting “pure” tonic seizures. Of note, we emphasize that none of the neonates with acute provoked seizures in our study had tonic seizures or even a tonic phase in the context of a sequential seizure.

## 5 STRENGTHS AND LIMITATIONS

This study provides novel data on electroclinical semiology of neonatal seizures among infants with NE in a sub‐Sahara African settings, where therapeutic hypothermia is not available. Evidence of perinatal hypoxia‐ischemia in the eligibility criteria was largely based clinically on the need for resuscitation after birth in combination with Apgar score, as fetal monitoring and blood gas measurement are not routinely performed. Although differences in the definition of perinatal hypoxia‐ischemia exist between regional and national jurisdictions around the globe,^30^ our NE study cohort may not therefore be fully comparable to HIE cohorts in HICs. The correlation between clinical grade, EEG background state, seizures and neurodevelopmental outcome in this cohort has also been evaluated and is subject for a separate paper.

The cEEG monitoring started at a median (IQR) age of 21.1 h (14.2‐29.8), due to time required for completing informed consent procedures with the research personnel available. Considering the now widely recognized temporal distribution of acute provoked seizures in neonatal HIE,^31^ we must regard that a number of seizures have likely occurred before the monitoring started and have been missed from our analysis. Although we can generate amplitude integrated EEG trends from the EEG data obtained, seizure annotation on the aEEG was not performed in our study and therefore the correlation with EEG‐detected seizures was not examined. A detailed comparison however would be interesting in view of the desirable implementation of neurophysiology monitoring models in this setting. Since seizures in our Ugandan neonates were mainly prolonged, and represented by high amplitude discharge, the correlation between EEG‐detected and aEEG‐detected seizures is likely high.

The different electroclinical phenotype encountered in this cohort compared to other HIC NE cohorts might be due to other causes of neonatal seizures such as hypoglycemia and electrolyte imbalance; in our cohort blood glucose and electrolytes levels were infrequently monitored. Furthermore, seizure semiology may have been modified by treatment administration in the patients with video‐EEG recorded seizures who received ASM loads before the monitoring started. In the absence of any clear guidelines for the optimal timing of ASM administration, based on evidence that ASM administration within 1 h of seizure onset potentially reduces the seizure burden^18^ we expected that seizures should be treated within 1 h of onset to be considered as having been appropriately treated. The generalizability of our results on seizure recognition and treatment appropriateness is limited by the fact that a dedicated group of research nurses looked after the study neonates, whereas the ratios of nurses to patients are typically lower in routine practice.

## 6 CONCLUSION

Retrospective review of continuous video‐EEG‐polygraphy monitoring in a cohort of neonates with NE in Uganda revealed a high incidence of neonatal seizures and status epilepticus. Many seizures were not treated, and non‐seizure events were often treated with ASM. The majority of patients in the seizure group presented with electroclinical seizures at some point, with clonic, autonomic and automatisms representing the more frequently encountered seizure types. Respiratory impairment emerged as a prominent concern, through both prolonged ictal apnea during the seizure and non‐ictal abdominal flutter or gasping without electrographic seizure. Enhanced knowledge of seizure semiology in this setting and improved availability of neurophysiology monitoring would help guide management.

## AUTHOR CONTRIBUTIONS

J. Proietti analyzed and interpreted the data and wrote the manuscript; S.R. Mathieson and S. Sadoo analyzed and interpreted the data; C. Nanyunja, E. Duckworth, I. Mambule and A. Nakimuli collected and verified the data; C.J. Tann conceived and designed the study, and collected and verified and interpreted the data; G.B. Boylan conceived and designed the study and analyzed and interpreted the data. All authors edited and approved the final draft of the manuscript.

## FUNDING INFORMATION

This work is supported by The Bill & Melinda Gates Foundation grant number OPP1210890. The sponsor is the MRC/UVRI and LSHTM Uganda Research Unit, Entebbe. This work was also supported by an Innovator award from the Wellcome Trust (209325/Z/17/Z). The funder had no role in the research design, or in the execution, analyses, interpretation of the data, or decision to submit results.

## CONFLICT OF INTEREST STATEMENT

GB Boylan is founder and shareholder in Kephala Ltd. and Cergenx Ltd. The remaining authors have no conflicts of interest.

## PARTICIPANT CONSENT STATEMENT

Written consent for video recording and data sharing was acquired from parents for all participants.

**Test yourself**

1. Retrospective analysis of continuous video‐EEG‐polygraphy monitoring in neonates with NE in Uganda revealed neonatal seizures:

A. In all patients

B. Only within the earliest 24 h after birth

C. In less than 20% of neonates monitored

D. In over 50% of neonates monitored

E. Which had been already identified at cot side based on clinical features only

2. Most patients with seizures in this Ugandan neonatal encephalopathy cohort presented with electroclinical seizures at some point; the more frequently encountered seizures types were:

A. Tonic seizures

B. Clonic, autonomic and automatisms seizures

C. Epileptic spasms and myoclonic seizures

D. Behavioral arrest seizures

E. Sequential seizures

3. Respiratory impairment:

A. Was common in neonates with autonomic seizures, often presenting with prolonged ictal apnea

B. Frequently went unrecognized by clinical staff

C. Emerged as a prominent concern in this setting with limited access to neonatal intensive care

D. All the previous

E. None of the previous

*Answers may be found in the* *supporting information*.

## Supporting information




Data S1.

## Data Availability

Further data that support the findings of this study are available on request from the corresponding author.

## References

[1] United Nations Inter‐Agency Group for Child Mortality Estimation (UN IGME) . Levels and trends in child mortality, report. 2022. Available at: https://data.unicef.org/resources/levels‐and‐trends‐in‐child‐mortality/

[2] Lee AC , Kozuki N , Blencowe H , Vos T , Bahalim A , Darmstadt GL , et al. Intrapartum‐related neonatal encephalopathy incidence and impairment at regional and global levels for 2010 with trends from 1990. Pediatr Res. 2013;74(Suppl 1):50–72. 24366463 10.1038/pr.2013.206 PMC3873711

[3] Tann CJ , Webb EL , Lassman R , Ssekyewa J , Sewegaba M , Musoke M , et al. Early childhood outcomes after neonatal encephalopathy in Uganda: a cohort study. Clin Med. 2018;6:26–35. 10.1016/j.eclinm.2018.12.001 PMC6358042 30740596

[4] Thayyil S , Pant S , Montaldo P , Shukla D , Oliveira V , Ivain P , et al. HELIX consortium. Hypothermia for moderate or severe neonatal encephalopathy in low‐income and middle‐income countries (HELIX): a randomised controlled trial in India, Sri Lanka, and Bangladesh. Lancet Glob Health. 2021;9(9):e1273–e1285. 34358491 10.1016/S2214-109X(21)00264-3 PMC8371331

[5] Kharoshankaya L , Stevenson NJ , Livingstone V , Murray DM , Murphy BP , Ahearne CE , et al. Seizure burden and neurodevelopmental outcome in neonates with hypoxic‐ischemic encephalopathy. Dev Med Child Neurol. 2016;58(12):1242–1248. 27595841 10.1111/dmcn.13215 PMC5214689

[6] Dilena R , Raviglione F , Cantalupo G , Cordelli DM , De Liso P , Di Capua M , et al. Consensus protocol for EEG and amplitude‐integrated EEG assessment and monitoring in neonates. Clin Neurophysiol. 2021;132(4):886–903. 33684728 10.1016/j.clinph.2021.01.012

[7] Murray DM , Boylan GB , Ali I , Ryan CA , Murphy BP , Connolly S . Defining the gap between electrographic seizure burden, clinical expression and staff recognition of neonatal seizures. Arch Dis Child Fetal Neonatal Ed. 2008;93(3):F187–F191. 17626147 10.1136/adc.2005.086314

[8] Nair B , Sharma J , Chaudhary S . Clinicoetiological profile of neonatal seizure in a newborn care unit of a tertiary care teaching hospital in northern India. J Clin Neonatol. 2020;9:27–31.

[9] Sabzehei MK , Basiri B , Bazmamoun H . The etiology, clinical type, and short outcome of seizures in newborns hospitalized in Besat hospital/Hamadan/Iran. Iran J Child Neurol. 2014;8:24–28. PMC4058061 24949047

[10] Mwaniki M , Mathenge A , Gwer S , Mturi N , Bauni E , Newton CRJC , et al. Neonatal seizures in a rural Kenyan district hospital: aetiology, incidence and outcome of hospitalization. BMC Med. 2010;8:16. 20236524 10.1186/1741-7015-8-16 PMC2846860

[11] Nunes ML , Yozawitz EG , Zuberi S , Mizrahi EM , Cilio MR , Moshé SL , et al. Neonatal seizures: is there a relationship between ictal electroclinical features and etiology? A critical appraisal based on a systematic literature review. Epilepsia Open. 2019;4(1):10–29. 30868112 10.1002/epi4.12298 PMC6398099

[12] Santarone ME , Pietrafusa N , Fusco L . Neonatal seizures: when semiology points to etiology. Seizure. 2020;80:161–165. 32585613 10.1016/j.seizure.2020.06.025

[13] Nanyunja C , Sadoo S , Mambule I , Mathieson SR , Nyirenda M , Webb EL , et al. Protocol for the Birth Asphyxia in African Newborns (Baby BRAiN) Study: a neonatal encephalopathy feasibility cohort study. Gates Open Res. 2022;3(6):10. 10.12688/gatesopenres.13557.1 PMC9110736 35614965

[14] Thompson CM , Puterman AS , Linley LL , Hann FM , van der Elst CW , Molteno CD , et al. The value of a scoring system for hypoxic ischaemic encephalopathy in predicting neurodevelopmental outcome. Acta Paediatr. 1997;86(7):757–761. 9240886 10.1111/j.1651-2227.1997.tb08581.x

[15] Clancy RR , Legido A . The exact ictal and interictal duration of electroencephalographic neonatal seizures. Epilepsia. 1987;28:537–541. 3653058 10.1111/j.1528-1157.1987.tb03685.x

[16] Lawrence R , Inder T . Neonatal status epilepticus. Semin Pediatr Neurol. 2010;17:163–168. 20727485 10.1016/j.spen.2010.06.010

[17] Pressler RM , Cilio MR , Mizrahi EM , Moshé SL , Nunes ML , Plouin P , et al. The ILAE classification of seizures and the epilepsies: modification for seizures in the neonate. Position paper by the ILAE Task Force on Neonatal Seizures. Epilepsia. 2021 Mar;62(3):615–628. 33522601 10.1111/epi.16815

[18] Pavel AM , Rennie JM , de Vries LS , Blennow M , Foran A , Shah DK , et al. Neonatal seizure management: is the timing of treatment critical? J Pediatr. 2022;243:61–68.e2. 34626667 10.1016/j.jpeds.2021.09.058 PMC9067353

[19] Bellos I , Devi U , Pandita A . Therapeutic hypothermia for neonatal encephalopathy in Low‐ and middle‐income countries: a meta‐analysis. Neonatology. 2022;119(3):300–310. 10.1159/000522317 35340015

[20] Low E , Boylan GB , Mathieson SR , Murray DM , Korotchikova I , Stevenson NJ , et al. Cooling and seizure burden in term neonates: an observational study. Arch Dis Child Fetal Neonatal Ed. 2012;97(4):F267–F272. 10.1136/archdischild-2011-300716 22215799

[21] Guidotti I , Lugli L , Guerra MP , Ori L , Gallo C , Cavalleri F , et al. Hypothermia reduces seizure burden and improves neurological outcome in severe hypoxic‐ischemic encephalopathy: an observational study. Dev Med Child Neurol. 2016;58(12):1235–1241. 10.1111/dmcn.13195 27444888

[22] Vegda H , Krishnan V , Variane G , Bagayi V , Ivain P , Pressler RM . Neonatal seizures‐perspective in low‐and middle‐income countries. Indian J Pediatr. 2022;89(3):245–253. 10.1007/s12098-021-04039-2 35050459 PMC8857130

[23] Nash KB , Bonifacio SL , Glass HC , Sullivan JE , Barkovich AJ , Ferriero DM , et al. Video‐EEG monitoring in newborns with hypoxic‐ischemic encephalopathy treated with hypothermia. Neurology. 2011;76(6):556–562. 21300971 10.1212/WNL.0b013e31820af91a PMC3053178

[24] Cornet MC , Morabito V , Lederer D , Glass HC , Ferrao Santos S , Numis AL , et al. Neonatal presentation of genetic epilepsies: early differentiation from acute provoked seizures. Epilepsia. 2021;62(8):1907–1920. 34153113 10.1111/epi.16957

[25] Pellegrin S , Munoz FM , Padula M , Heath PT , Meller L , Top K , et al. Neonatal seizures: case definition & guidelines for data collection, analysis, and presentation of immunization safety data. Vaccine. 2019;37(52):7596–7609. 31783981 10.1016/j.vaccine.2019.05.031 PMC6899436

[26] Ramírez JD , Gonzales M , Hoyos JA , Grisales L . Diaphragmatic flutter: a case report and literature review. Neurologia. 2015;30(4):249–251. 24011669 10.1016/j.nrl.2013.06.016

[27] Malone A , Ryan CA , Fitzgerald A , Burgoyne L , Connolly S , Boylan GB . Interobserver agreement in neonatal seizure identification. Epilepsia. 2009;50(9):2097–2101. 10.1111/j.1528-1167.2009.02132.x 19490044

[28] Variane GFT , Dahlen A , Pietrobom RFR , Rodrigues DP , Magalhães M , Mimica MJ , et al. Remote monitoring for seizures during therapeutic hypothermia in neonates with hypoxic‐ischemic encephalopathy. JAMA Netw Open. 2023;6(11):e2343429. 10.1001/jamanetworkopen.2023.43429 37966836 PMC10652158

[29] de Corrêa NC , Bom JMDS , Scherer MR , Nunes ML . Clinical profile of a cohort of neonates with seizures: association between semiology, etiology, and electroencephalographic findings. Pediatr Neonatol. 2022;63(6):582–589. 35922262 10.1016/j.pedneo.2022.04.009

[30] Proietti J , Boylan GB , Walsh BH . Regional variability in therapeutic hypothermia eligibility criteria for neonatal hypoxic‐ischemic encephalopathy. Pediatr Res. 2024. 10.1038/s41390-024-03184-6 PMC11521984 38649726

[31] Pavel AM , Rennie JM , de Vries LS , Mathieson SR , Livingstone V , Finder M , et al. Temporal evolution of electrographic seizures in newborn infants with hypoxic‐ischaemic encephalopathy requiring therapeutic hypothermia: a secondary analysis of the ANSeR studies. Lancet Child Adolesc Health. 2024;8(3):214–224. 38246187 10.1016/S2352-4642(23)00296-1 PMC10864190

