# Supplementary material for: Retrospective characterization of seizure semiology and treatment using continuous video‐EEG monitoring in neonatal encephalopathy in Uganda
Source: Epileptic Disord. 2024 Nov 18;27(1):31–43. doi: 10.1002/epd2.20299 (PMC11829619; doi:10.1002/epd2.20299)
Supplement: Supplementary file 1 — Data S1. [file EPD2-27-31-s001.docx]

Test yourself.

Answers:

(1) D

(2) B

(3) D
